# The potential of articaine as new generation of local anesthesia in dental clinics: A review

**DOI:** 10.1097/MD.0000000000032089

**Published:** 2022-12-02

**Authors:** Wen Luo, Kaiyue Zheng, Huifang Kuang, Zhixin Li, Jinrong Wang, Jie Mei

**Affiliations:** a Department of Stomatology, the First Affiliated Hospital of Hainan Medical University, Haikou, China; b School of Stomatology, Hainan Medical University, Haikou, China

**Keywords:** articaine, dental treatment, lidocaine, local anesthesia

## Abstract

As a new drug for local dental anesthesia, articaine has become popular in the clinic in recent years. In this review, we describe the development of articaine, explain its mechanism of action, compare its efficacy with that of other commonly used local anesthetics in dental treatment, and summarize the application of articaine in special populations. In conclusion, the anesthetic efficacy of articaine in clinical dental treatment is better than that of lidocaine, and its safety is not statistically different from that of lidocaine. In particular, articaine has several advantages and can be selected flexibly for clinical use. Atecaine has great potential for wide application in dental clinics in the near future.

## 1. Introduction

Pain control in dental clinics is mainly achieved using local anesthetics. A good anesthetic effect can reduce pain and discomfort, improve patients’ cooperation, and reduce patient anxiety.^[1,2]^ Articaine is currently an anesthetic used in dental clinics. Articaine was first synthesized and named carticaine in 1969.^[3]^ Winther and Nathalang conducted the first clinical trial in Germany in 1971.^[4]^ In 1984, the name was changed from carticaine to articaine, and it was used in Canada.^[5]^ In 2006, the US Food and Drug Administration (FDA) approved 4% articaine plus 1:200,000 adrenaline solution for clinical use^.[6]^ At present, 4% articaine plus 1:100,000 or 1:200,000 adrenaline solutions are commonly used in clinical practice^.[7,8]^

**Figure 1.**
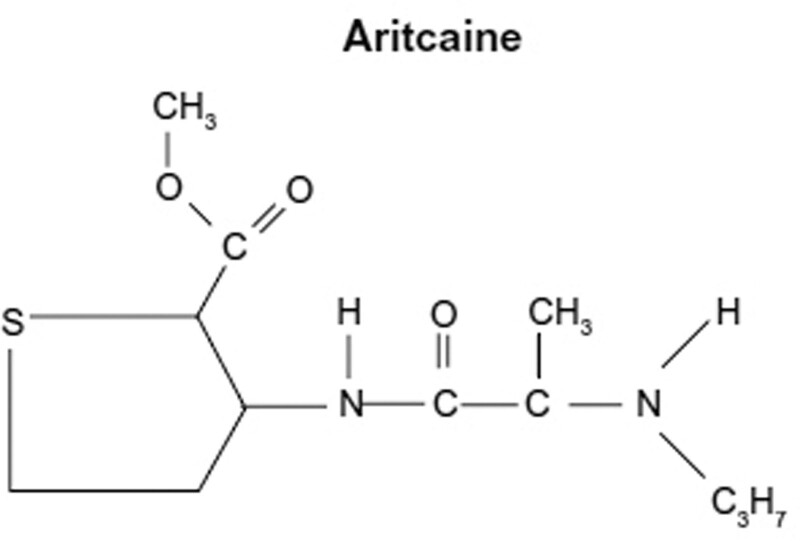
The chemical structure of articaine.

The chemical name of articaine was 4-methyl-3-[[1-oxo-2-(propylamino)-propyl]amino]-2-thiophene carboxylic acid methyl ester hydrochloride. Articaine is a unique amide compound that contains a thiophene rather than a benzene ring (Fig. 1).^[2,9]^ The thiophene ring allows greater liposolubility and potency; therefore, a larger proportion of the administered dose can enter neurons to block ion channels.^[10]^

**Figure 2.**
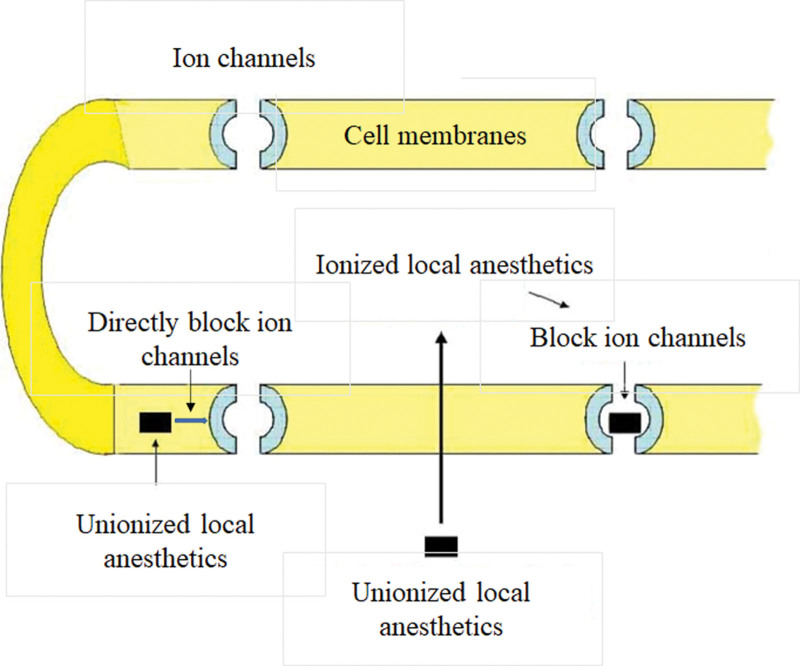
The mechanism of action of local anesthetics including articaine.

Local anesthetics disrupt the function of ion channels in the cell membranes of neurons to prevent the transmission of action potentials. The underlying mechanism is that local anesthetic molecules in the unionized form cross the cell membrane to enter the cytoplasm to become ionized and then specifically bind to sodium channels to keep them in an inactive state and prevent depolarization. In addition, the unionized form of local anesthetic molecules can be directly incorporated into the cell membranes without entering the cytoplasm or disrupting the function of ion channels on the cell membranes (Fig. 2).^[11,12]^ Nerve fibers show different sensitivities to local anesthetics. Although small nerve fibers are usually more sensitive to local anesthetics than large nerve fibers, myelinated nerve fibers are blocked faster than non-myelinated nerve fibers. Therefore, loss of nerve function proceeds as a loss of pain, temperature, touch, proprioception, and skeletal muscle tone. This could explain why we felt touch but not pain after administration of local anesthesia^.[13,14]^

Articaine is the only amide local anesthetic that contains a thiophene ring and an additional ester ring.^[6]^ Dental clinicians prefer to use amide local anesthetics because amide drugs can achieve the effect of surgical anesthesia faster and more reliably and have fewer allergic reactions than ester anesthetics.^[15,16]^ However, whether articaine is safe and effective in specific groups such as the elderly, children and pregnant women is still controversial. Therefore, we selected “Articaine”; “Lidocaine”; “Local anesthesia”; “dental treatment” as key words to search the relevant articles published in PubMed, Web of Science and Embace databases from 1975 to 2022, excluding duplicate articles, a total of 323 articles were searched, screened, and summarized, and finally 53 related articles were included in this review.

### 1.1. Comparison of articaine with other commonly used local anesthetics in dental treatment

Lidocaine has been proved to be a safe and effective local anesthetic. Since lidocaine entered the clinic in 1948, it has become the most common local dental anesthetic in most countries.^[17]^ Lidocaine has been established as the gold standard for dental local anesthesia, and all new local anesthetics must be compared with it.^[10,18]^ The main characteristics of lidocaine and articaine are summarized in Table 1.^[19–21]^ In dental clinical applications, a small amount of adrenaline injection is usually added to lidocaine local anesthetic to reduce bleeding and blood oozing during the operation. However, it is difficult to master the standard preparation dose during clinical operations, and secondary pollution can easily occur.^[22]^

**Table 1:** Main characteristics of lidocaine and articaine.

	lidocaine	articaine
Onset time	Infiltration anesthesia: 1–3 min Block anesthesia: 5 min	4 min
Duration	Infiltration anesthesia: 120 min Block anesthesia:120–150 min	About 144 min
Adult dosage	Infiltration anesthesia: <4.5 mg/kg Block anesthesia: <4.0 mg/kg	≤7 mg/kg
Children dosage	<4.0–4.5 mg/kg	≤5 mg/kg
neurotoxicity	Moderate	Low
Adverse reactions	Rarely occur	Rarely occur
Precautions	(1)It is highly toxic and easy to be absorbed and should be used with caution for infiltration anesthesia. (2)It is easy to pass through the placenta and should be used with caution for pregnant women.	(1)It is forbidden for patients with hypertension and children under 4 years old. (2)It should be used with caution for patients with diabetes and patients used with monoamine oxidase inhibitors

Articaine is packaged with a pre-installed cartridge and equipped with a special syringe, which can avoid risks such as secondary pollution, and the injection needle is thin, which can reduce patient pain during anesthetic injection.^[23]^ Martin et al showed that the success rate of lidocaine in infiltration anesthesia was 2.78 times that of lidocaine (*P* = .0002), of which the success rate in mandibular infiltration anesthesia was 3.01 times that of lidocaine, and the success rate in maxillary infiltration anesthesia was 2.61 times that of lidocaine (*P* = .01).^[24]^ Ashraf et al showed that for teeth requiring root canal treatment, when buccal supplementary anesthesia was performed after mandibular block anesthesia failed, the success rate of articaine in anesthesia was 71%, and the success rate of lidocaine in anesthesia was 29% (*P* < .001). The results confirmed that the effect of articaine on supplementary buccal infiltration anesthesia was significantly better than that of lidocaine.^[25]^ The molecular structure of articaine includes a thiophene ring and ester side chain. When articaine is absorbed into the systemic circulation from the injection site, it is rapidly inactivated by the hydrolysis of the ester side chain. Therefore, articaine has the shortest metabolic half-life (estimated to be 27–42 min), whereas the elimination half-life of most amide local anesthetics, such as lidocaine, is 90 min. It is believed that articaine is less toxic than lidocaine.^[15,26]^ No serious adverse reactions were reported during the clinical application of articaine, and minor adverse events included postoperative pain, headache, facial edema, infection, gingivitis, and temporary paresthesia.^[27]^ These effects were similar to those of lidocaine with a similar frequency. Studies have shown that the total incidence of adverse reactions to 4% articaine plus 1:100,000 adrenaline was 2.2%, while that of 2% lidocaine plus 1:100,000 adrenaline was 2.0%.^[28]^ In Canada, articaine surpassed lidocaine as the most commonly used dental anesthetic.^[15]^

Bupivacaine was developed by Ekenstam, Egner, and Pettersson in 1957 and was first used clinically in 1964.^[29]^ Bupivacaine is an amide anesthetic and a so-called “long-acting” local anesthetic with a long duration of action and residual analgesia.^[30]^ The long-acting anesthetic and analgesic effects of bupivacaine make the operation comfortable, but prolonged anesthesia in soft tissues is uncomfortable for patients.^[31]^ It has been reported that articaine has better clinical effects than bupivacaine, with shorter latency, less bleeding, shorter soft tissue anesthesia time, better anesthesia efficacy, and lower anesthetic dose than bupivacaine.^[29]^ Therefore, it is recommended to consider the use of bupivacaine in long-term surgery or surgery, which is expected to cause severe pain early after surgery. Articaine is preferred in terms of anesthetic efficacy, intraoperative comfort, and solution dosage.

Mepivacaine is an amide anesthetic that can be used alone or in combination with adrenaline. The anesthetic efficacy of mepivacaine is intermediate to that of articaine and lidocaine. Mepivacaine without adrenaline can be used as the preferred local anesthetic for the elderly or patients with cardiovascular diseases because it does not contain a vasoconstrictor. A study on the extraction of maxillary teeth under local anesthesia with articaine and mepivacaine in 94 volunteers showed that buccal injection of articaine had a shorter onset time and better efficacy than mepivacaine.^[32]^ The mean onset time of maxillary pulpal anesthesia was 2.98 min, which was significantly shorter than 4.22 min in the mepivacaine group.

### 1.2. Application of articaine in dental treatment for special populations

#### 1.2.1. Application of articaine in children.

Pain control is the most important aspect of pediatric dentistry. Inferior alveolar nerve block is one of the most painful injection methods for local dental anesthesia in children.^[33–35]^ Articaine is used in local infiltration anesthesia, with a small injection needle and a small injection volume, and achieves good efficacy in avoiding pain.^[36,37]^ Articaine should be avoided in children aged 0 to 4 years original manufacturer’s instructions.^[38]^ However, the most common guideline for the use of articaine by the American Academy of Pediatric Dentistry (2015) did not state that articaine cannot be used in children under the age of 4, or it increases the risk of soft tissue trauma.^[18]^ Gulenko et al proved the efficacy of articaine in children under 4 years of age, and it was considered a safe substitute for lidocaine, which can be used in children of all ages.^[39]^ At present, the recommended dose of articaine for children is 5 mg/kg, and this dose should be evaluated and calculated during clinical use. Although new studies have proved that articaine can be used in children under 4 years old, it is suggested that children under 4 years old should be used with caution, but it is not a contraindication.

#### 1.2.2. Application of articaine in the elderly.

Oertel et al investigated the effects of age on the pharmacokinetics and pharmacodynamics of articaine. The concentration of articaine in the serum was determined by HPLC, and pharmacokinetic parameters were calculated using a double exponential equation according to the standard procedure. Compared to young healthy volunteers, the clearance rate and distribution volume of articaine in the elderly were lower. However, there was no significant difference between young and old volunteers in the blood concentration-time curve area, maximum drug concentration, drug end half-life, or time to reach the maximum blood concentration.^[40]^ Other studies have shown that articaine metabolism is independent of age, and there is no need to change the dose in the elderly population. However, the dose should be reduced as appropriate for the elderly with certain underlying diseases.

#### 1.2.3. Application of articaine in pregnant women.

Pregnant women may experience dental emergency symptoms such as acute pulpitis and wisdom tooth pericoronitis, which cause great pain and anxiety in pregnant women and adversely affect fetal growth and development. Timely painless dental treatment is very important. Stomatologists are more concerned about the safety and effectiveness of local anesthetics. According to the index classification of drug safety during pregnancy formulated by the US Food and Drug Administration, local anesthetics used in stomatology in the form of injections are divided into 2 categories: lidocaine (class B). Animal studies have shown that it has no adverse effects on the fetus, but there are no sufficient and strict control studies on pregnant women. Articaine and mepivacaine (Class C). Animal studies have shown that they have adverse effects on the fetus, and there are no sufficient and strict control studies on pregnant women. Although there may be risks, the potential efficacy of these drugs in pregnant women may justify their use.^[39]^ Anisimova et al introduced the latest guidelines for emergency dental care in pregnant women and recommended the use of 4% articaine plus 1:200,000 adrenaline as the first-choice drug for local anesthesia in pregnant women.^[41]^ However, it should be noted that its use is not recommended in the first three months and the last three months of pregnancy.

### 1.3. Application of articaine in clinical departments of stomatology

#### 1.3.1. Application of articaine in endodontics.

In a study on the effects of different anesthetic drugs on the efficacy of inferior alveolar nerve block in the treatment of irreversible pulpitis, de Geus et al found that the success rate of articaine was 73%, the success rate of prilocaine was 57%, the success rate of mepivacaine was 55%, while the success rate of lidocaine was only 12%; for patients with irreversible pulpitis, articaine had a better analgesic effect.^[42]^ In a case study of 746 patients with irreversible pulpitis who had persistent pulpal pain after successful mandibular block anesthesia, Peters et al found that the success rate of auxiliary infiltration anesthesia with articaine instead of lidocaine was 3.55 times that of general anesthesia and no adverse events occurred.^[43]^ Nagendrababu et al also confirmed in the latest evidence-based medicine statistics that the local anesthetic effect of articaine is more effective in root canal treatment of irreversible pulpitis, and the injection of articaine has less pain, faster effect and less adverse reactions.^[44]^ Therefore, articaine has more advantages in the treatment of dental pulp.

#### 1.3.2. Application of articaine in oral and maxillofacial surgery.

The safety and effectiveness of local anesthetics for suppurative inflammatory tissues in the maxillofacial region have been a major concern for many dentists. In a study on tumescent local anesthesia, Grossmann et al found that articaine had low toxicity to the central nervous system and caused low allergy in tissues, and its use for local anesthesia in tumescent areas was safe.^[45]^ In terms of effectiveness, the pKa (dissociation constant of local anesthetic molecules) value of articaine was 7.8, similar to the pH value (7.4) of intact human tissues, and it had the effects of rapid hydrolysis and rapid anesthesia. In local infiltration technology anesthesia can be achieved after 1–2 minutes, and in nerve retardation anesthesia anesthesia can be achieved after 2–5 minutes . In inflammatory tissues, hydrolysis in an acidic environment is a serious problem and the anesthetic effect worsens. Compared with other amide local anesthetics, articaine is more effective for anesthesia of suppurative inflammatory tissues.^[46]^ A meta-analysis on the efficacy and safety of articaine in the extraction of mandibular third molars showed that the use of 4% articaine for the extraction of mandibular third molars was a safer option than other amide local anesthetics, required less dose, and had a shorter onset time.^[47]^ Oertel et al suggested that articaine should be selected for local anesthesia in outpatient surgery of oral and maxillofacial surgery.^[40]^

#### 1.3.3. Application of articaine in implantology.

The development of stem cells have raised hope for dental implantology.^[48,49]^ In the past, using lidocaine for nerve block anesthesia was the main anesthesia method in oral implantology; however, the onset time of anesthesia was long, the failure rate of anesthesia was high, and it was difficult to control the extent of anesthesia. If the inferior alveolar nerve is damaged during the operation, the patient has no pain response and cannot provide timely feedback to the operator, which increases the risk of surgery. In a study of postoperative pain and swelling in 100 implantation patients, Sánchez-Siles et al concluded that excessive local anesthetic injection during implant surgery had a negative impact on postoperative pain and swelling, as well as patient satisfaction.^[50]^ In a randomized controlled trial, Moaddabi et al found that both systolic and diastolic blood pressure of lidocaine and articaine increased after local infiltration anesthesia. however, there was no significant difference between the two drugs in increasing blood pressure.^[51]^ Studies have shown that articaine is safe and reliable and can be used as a substitute for lidocaine in local infiltration anesthesia. Articaine has the shortest metabolic half-life, and high-dose injections can be used to significantly reduce post-implantation reactions.

### 1.4. Adverse reactions of articaine and lidocaine.

Yamashita et al showed that the most common adverse reactions of articaine and lidocaine were sweating and pallor, followed by dizziness, palpitation, tremor and hypertension. The adverse reaction rate of lidocaine group was 3.85%, while that of articaine group was 3.60%. Both of them were safe in oral local anesthesia. The occurrence of these local adverse reactions may be related to the local anesthetic containing epinephrine, and the local ischemia at the injection site is caused by the compactness of the injection site.^[52]^ This suggests that we should strictly control the dosage, concentration and speed of injection, and the speed should be less than 1 mL/min. There is no significant difference in adverse reactions between articaine and lidocaine. Reports of lidocaine allergy are rare, but Dey et al reported a patient with lidocaine allergy. After the allergy to articaine was excluded by skin sensitivity test, the affected teeth of the patient were extracted after local anesthesia with articaine, the report suggested that articaine can be used as a suitable choice for patients with lidocaine allergy.^[53]^

## 2. Conclusions

In summary, articaine has the advantages of low toxicity, good local infiltration and high biological safety. Articaine has a higher success rate in dental infiltration anesthesia and reduces the pain of patients. Articaine can be used as a good choice for local anesthetics for routine dental treatment with application potential in dental clinic in the near future.

## Author contributions

**Conceptualization:** Wen Luo.

**Data curation:** Kaiyue Zheng.

**Formal analysis:** Kaiyue Zheng.

**Investigation:** Kaiyue Zheng, Huifang Kuang.

**Writing—original draft:** Zhixin Li, Jinrong Wang.

**Writing—review and editing:** Jie Mei.
